# Diagnosing Hirschsprung disease by detecting intestinal ganglion cells using label-free hyperspectral microscopy

**DOI:** 10.1038/s41598-021-80981-7

**Published:** 2021-01-14

**Authors:** Marcos A. Soares de Oliveira, Laura Galganski, Sarah Stokes, Che -Wei Chang, Christopher D. Pivetti, Bo Zhang, Karen E. Matsukuma, Payam Saadai, James W. Chan

**Affiliations:** 1 Department of Pathology and Laboratory Medicine, University of California Davis, Sacramento, CA 95817 USA; 2 Division of Pediatric General, Thoracic and Fetal Surgery, University of California Davis, Sacramento, CA 95817 USA; 3 Shriners Hospitals for Children, Pediatric Colorectal Center, Sacramento, CA 95817 USA

**Keywords:** Imaging, Microscopy, Optical spectroscopy, Gastrointestinal diseases, Biomedical engineering, Imaging and sensing, Microscopy, Optical spectroscopy, Raman spectroscopy, Optics and photonics, Biophotonics

## Abstract

Hirschsprung disease (HD) is a congenital disorder in the distal colon that is characterized by the absence of nerve ganglion cells in the diseased tissue. The primary treatment for HD is surgical intervention with resection of the aganglionic bowel. The accurate identification of the aganglionic segment depends on the histologic evaluation of multiple biopsies to determine the absence of ganglion cells in the tissue, which can be a time-consuming procedure. We investigate the feasibility of using a combination of label-free optical modalities, second harmonic generation (SHG); two-photon excitation autofluorescence (2PAF); and Raman spectroscopy (RS), to accurately locate and identify ganglion cells in murine intestinal tissue without the use of exogenous labels or dyes. We show that the image contrast provided by SHG and 2PAF signals allows for the visualization of the overall tissue morphology and localization of regions that may contain ganglion cells, while RS provides detailed multiplexed molecular information that can be used to accurately identify specific ganglion cells. Support vector machine, principal component analysis and linear discriminant analysis classification models were applied to the hyperspectral Raman data and showed that ganglion cells can be identified with a classification accuracy higher than 95%. Our findings suggest that a near real-time intraoperative histology method can be developed using these three optical modalities together that can aid pathologists and surgeons in rapid, accurate identification of ganglion cells to guide surgical decisions with minimal human intervention.

## Introduction

Hirschsprung disease (HD) is a congenital intestinal motility disorder affecting 1 in 5000 new babies in the United States annually^1^. Children with HD lack vital intestinal nerve cells, or ganglion cells, which are responsible for normal motility and the ability to stool. The absence of ganglion cells (aganglionosis) leaves the affected portion of the intestine paralyzed and unable to propagate stool. If not properly diagnosed and treated, children can develop severe abdominal distention, vomiting, inability to feed, malnutrition, or death. HD mortality rates up to 20% have been reported. The proper management of HD requires the accurate histological localization of ganglion cells at the interface of the normal and diseased bowel. Then, through a pull-through surgical procedure, the aganglionic segment of the intestine is removed and the remaining healthy intestine is attached to the anus.

Currently, hematoxylin and eosin (H&E) histopathology is the ‘gold-standard’ for intraoperative HD diagnosis and requires surgical biopsies at multiple levels of the bowel to identify the extent of abnormal intestine. This process can be time-consuming and technically and diagnostically challenging as it requires multiple processing steps (e.g., tissue freezing, sectioning and staining) which introduce tissue artifacts and extend anesthesia and intra-operative time^2,3^. Furthermore, if the intraoperative findings are inconclusive or misinterpreted, a child may undergo excessive removal of healthy bowel^4^. Most devastatingly, a child may undergo incomplete resection of the aganglionic segment resulting in unrecognized, ongoing morbidity and necessitating reoperation, sometimes months to years later^5^.

Despite advances in the understanding of HD in the last few decades, the ability to identify this condition still mainly depends on the use of routine H&E stains and a pathologist’s experience and expertise in identifying ganglion cells. Ancillary to H&E histology, numerous histochemical and immunohistochemical staining techniques, such as acetylcholinesterase (AChE) and calretinin, have emerged but none of them have still been effectively established in routine intraoperative diagnoses mostly because of their lengthy processing times^6,7^. Patient management could be significantly improved if a reliable, real-time intraoperative diagnostic method was available that could more rapidly and accurately detect intestinal ganglion cells. Such an approach could significantly reduce the overall time the patient is under anesthesia, improve clinical outcomes, reduce the overall morbidity of HD, and lower overall healthcare costs.

Recent studies have demonstrated that label-free optical imaging modalities, such as multiphoton and Raman^8–11^, are promising approaches for near real-time histology in which the specimen no longer needs to be physically sectioned or stained. Multiphoton modalities, such as two-photon excitation autofluorescence (2PAF) and second harmonic generation (SHG), are well suited for high-resolution label-free molecular imaging. The native autofluorescence, from endogenous fluorophores in biological specimens, generated via two-photon excitation have been used as an image contrast for obtaining morphological, structural and also chemical information for a broad range of applications, such as real-time clinical diagnosis of oncological and other diseases^12–14^. SHG enables direct imaging of anisotropic biological structures, such as collagen^15^, by using the frequency doubled optical signal for the image contrast. These imaging modalities are simple to implement simultaneously with the proper choice of optical filters and detector placement. Raman spectroscopy is also a powerful label-free technique based on inelastic scattering of light by vibrational modes of molecular bonds. Because Raman spectroscopy excites multiple bonds associated with different molecules (e.g. protein, lipids, amino acids, nucleic acids), the optical signatures in a Raman spectra can provide a wealth of multiplexed chemical information for accurate identification and discrimination of biological specimens^16,17^. The large amount of information contained in the spectral data has been vastly exploited by using chemometric (multivariate mathematic and statistic) techniques such as principal component analysis (PCA) and linear discriminant analysis (LDA) to provide classification and quantification of the multidimensional spectral data. Additionally, in the last few decades, with the growth of artificial intelligence, chemometric methods, such as support vector machine (SVM) that enable computers to “learn” from a data base and to detect patterns from complex data sets, have also been widely used to assist in clinical decision making^18,19^.

The goal of this study was to determine whether the combination of 2PAF, SHG, and Raman scattering signals that were generated from intestinal tissues of C57/BL6 wild type mice could be used with chemometric methods to locate and identify ganglion cells in the tissue. Establishing the unique optical signals of ganglion cells in colon tissue is the first step in developing a new label-free intraoperative histology method for diagnosing HD. In this study, to ensure the optical signatures are assigned accurately to the ganglion cell and other regions of the tissue, detailed experiments were carried out in which label-free images were directly correlated to H&E images and the classification results based on the label-free spectral data were compared to the diagnosis by a pathologist.

## Methods and materials

### Sample preparation

**Figure 1:**
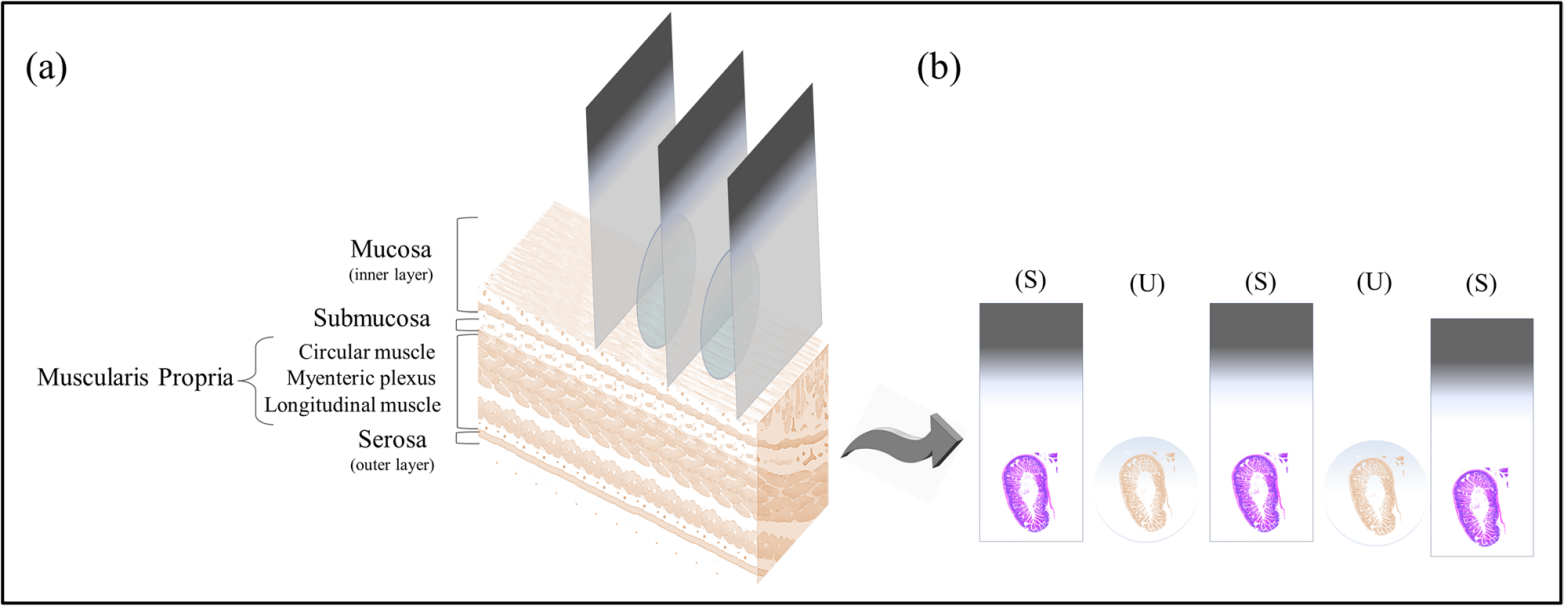
(**a**) Illustration of intestinal tissue demonstrating the multiple layers of the colon, from the serosa (outer) to the mucosa (inner). (**b**) H&E staining was used in every other tissue section of 5 µm thickness to identify ganglion cells and correlate with the adjacent unstained tissue.

All animal use and welfare in these studies adhered to the National Institutes of Health Guide for the Care and Use of Laboratory Animals following protocols reviewed and approved by the Institutional Animal Care and Use Committee (IACUC) of the University of California, Davis under IACUC Protocol #20942. These studies also comply with ARRIVE guidelines. Colon from 3-week-old C57/BL6 wild type mice were used in this study. Adjacent tissue sections of full thickness colon were prepared to allow for direct comparison of the label-free SHG/2PAF/Raman multimodal images with the H&E images, which serve as the ‘gold standard’ method for identifying ganglion cells. Figure 1a is an illustration of the cross section of the intestine showing different layers. The ganglion cells form the network of nerves called the myenteric plexus (Auerbach’s plexus) that are located in between the circular and the longitudinal muscle layers of the gastrointestinal (GI) tract wall.

Tissues dissected from the intestine were immersed in formalin for fixation. Tissues were then dehydrated by immersion in increasing concentrations (70, 80, 90 and 100%) of ethanol in water and embedded in melted paraffin. After the paraffin block was cooled to 4 °C in a fridge, serial 5 µm thick slices were cut such that each section contained all layers of intestine. Because ganglion cells are large enough to span multiple adjacent 5 µm sections, our method of sample preparation allows for comparison of standard H&E histopathology and label-free imaging from adjacent near-identical samples. To accomplish this, sections were deparaffinized by immersion in xylene and rehydrated in decreasing concentrations (100, 95, 70, 50 and 0%) of ethanol in water, and alternately stained (S) with H&E or left unstained (U), Fig. 1b, for label-free SHG/AF/Raman imaging without any further treatment. The unstained samples were prepared on quartz coverslips of #1 thickness.

### SHG and 2PAF microscopy

**Figure 2:**
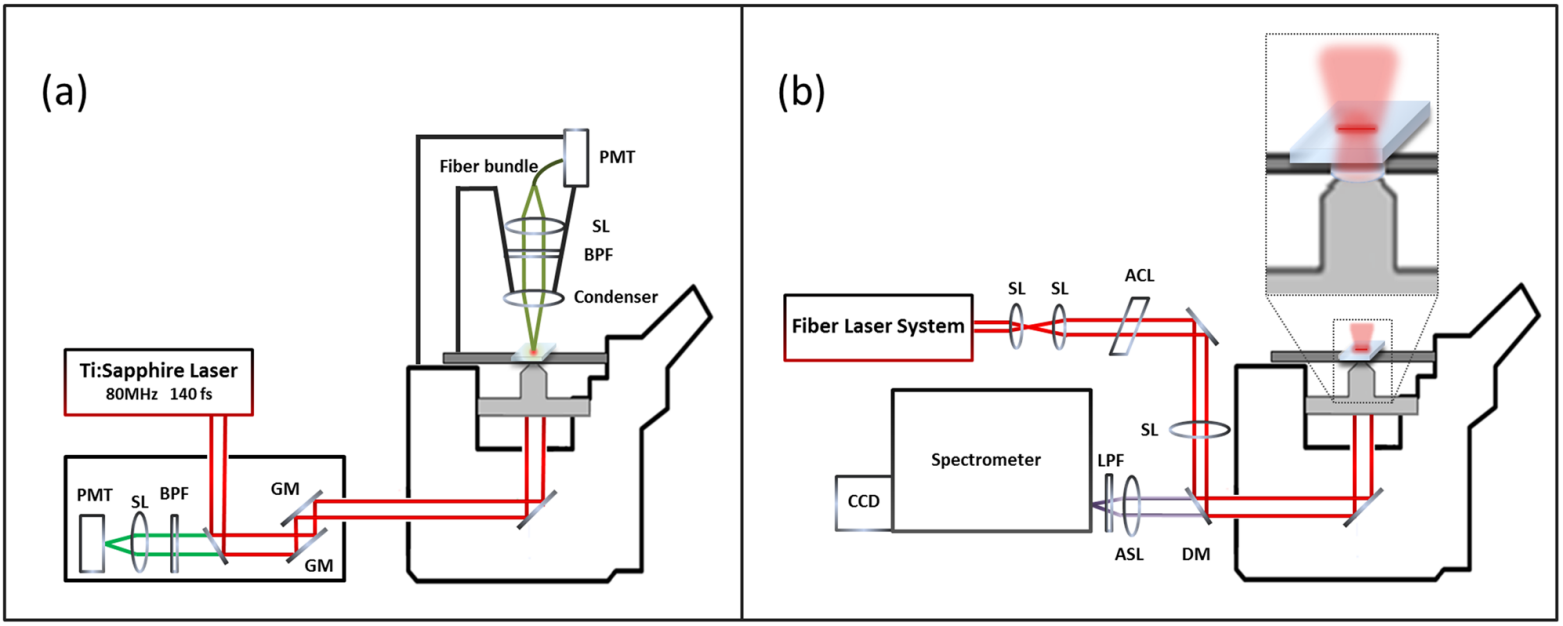
Experimental microscopy systems: (**a**) SHG and 2PAF; (**b**) Hyperspectral Raman. *ACL* achromatic cylindrical lens, *ASL* achromatic spherical sens, *BPF* bandpass filter, *DM* dichroic mirror, *GM* galvomirror, *LPF* longpass filter, *PMT* photomultiplier tube, *SL* spherical lens.

A laser scanning confocal microscope (Olympus Fluoview 300) was used for simultaneous 2PAF and SHG imaging^20^, Fig. 2a. Briefly, a Ti:Sapphire tunable (λ = 750–1000 nm) femtosecond laser, with a temporal resolution ~ 140 fs, was used as the excitation source. With an excitation wavelength of λ = 940 nm, the laser beam was focused into the sample with a 40X air objective (N.A. = 0.9). The laser power after the objective was ~ 20 mW. 2PAF signals were collected in the backward propagating direction by using a broad bandpass filter (BPF) at λ = 510–590 nm with a photomultiplier tube (PMT). SHG signals were collected through the forward direction with a 0.55 N.A. condenser lens and directed into another PMT with a BPF centered at 470 nm (λ/2). The typical scanning speed was 10 µs/pixel, making a total imaging time of 2.62 s/image for a 512 × 512 pixels image.

### Hyperspectral Raman microscopy

Hyperspectral Raman images of tissues were acquired using a separate Raman microscope system, Fig. 2b, as previously described^21^. Briefly, a fiber laser system (Sacher-Laser) with a wavelength of 785 nm was used as the excitation source. The laser beam passes an achromatic cylindrical lens (ACL) (Thorlabs, f = 100 mm) that focuses the Gaussian beam into a line profile and then is imaged by an achromatic spherical lens (ASL) (Thorlabs, f = 500 mm) onto the back aperture of a 60×, 1.2 N.A. water immersion objective lens (Olympus, UPlanSApo). The focused laser line in the sample plane has dimensions of ~ 1 by 50 µm. Raman signals from the focused laser line in the sample focal plane were imaged onto the entrance slit of the spectrometer (PI Acton, SpectraPro SP2300i) and collected by a CCD detector (PI Acton, Pixis100). The tissue sample sits on a motorized flat top translational stage (ProScan Prior II) of an inverted microscope (Leica, DM IRM), which scans the specimen relative to the laser focus. Typical Raman images were acquired using 30 s of integration time per line and a 1 µm step size in the direction perpendicularly to the long axis of the laser line focus.

### Data analysis

Brightfield images from both unstained and H&E stained slides were precisely overlaid with SHG, 2PAF and Raman images by using similar morphological features in the field of view as guides and used for further analysis and comparison.

Instead of using spectral signals from only a single Raman image pixel, signals from areas of 2 by 2 image pixels were averaged to produce a single Raman spectrum, which increases the signal-to-noise ratio (SNR) and yields more reproducible data while still retaining high spatial resolution. Background removal and normalization with respect to the total area under the curve were performed on each Raman spectrum. This processed spectrum was then used in all subsequent chemometric analyses. All data analyses were performed using custom Matlab algorithms (Mathworks, USA).

Two multivariate statistical analysis techniques, PCA and LDA, were used to build one of the two classification models in this study, as previously described^21^. PCA is an unsupervised method for identifying the combination of Raman spectral features that maximize the data variance. These features are captured in a new set of variables called principal components (PCs) in a reduced dimension. The first few PCs typically account for the majority of the data variance. However, as an unsupervised method, PCA has no prior knowledge about the groupings of the spectral data, which means it is not suitable for the purposes of group separation. LDA is a supervised technique and is useful for discriminating between groups. For optimizing group classification, a PCA-LDA model was developed in which the first three PCs were used as the input variables for LDA. A ‘leave-N-out’ cross-validation technique was used to test the classification sensitivity and specificity of the PCA-LDA model for different optical fingerprints in the tissue. This procedure involves taking all K-N (N = 10% of K by default) cells as a training set to build the LDA model, which is then used to classify the N ‘blind’ cells that were left out. This is done repeatedly for every possible group of N in the set of K cells. The accuracy of a prediction cross validation method of Raman spectra was presented by using the confusion matrix, as true negative (TN), false positive (FP), true positive (TP) and false negative (FN) classifications. Diagnostic accuracy, sensitivity and specificity were calculated.

The second classification model used in this study is based on SVM, which is a method of discrimination commonly used for pattern recognition in machine learning technology^18^. SVM is a supervised learning model with associated learning algorithm that analyzes data used for classification. The SVM training-based algorithm builds a model, by using a previous known set of data. The SVM model is a representation of the data as points in space, so any new data can be mapped into that same space and predicted to belong to a category based on which group they fit better. The prediction of this model was then presented by labeling any tested Raman fingerprint with its associated classification.

## Results

**Figure 3:**
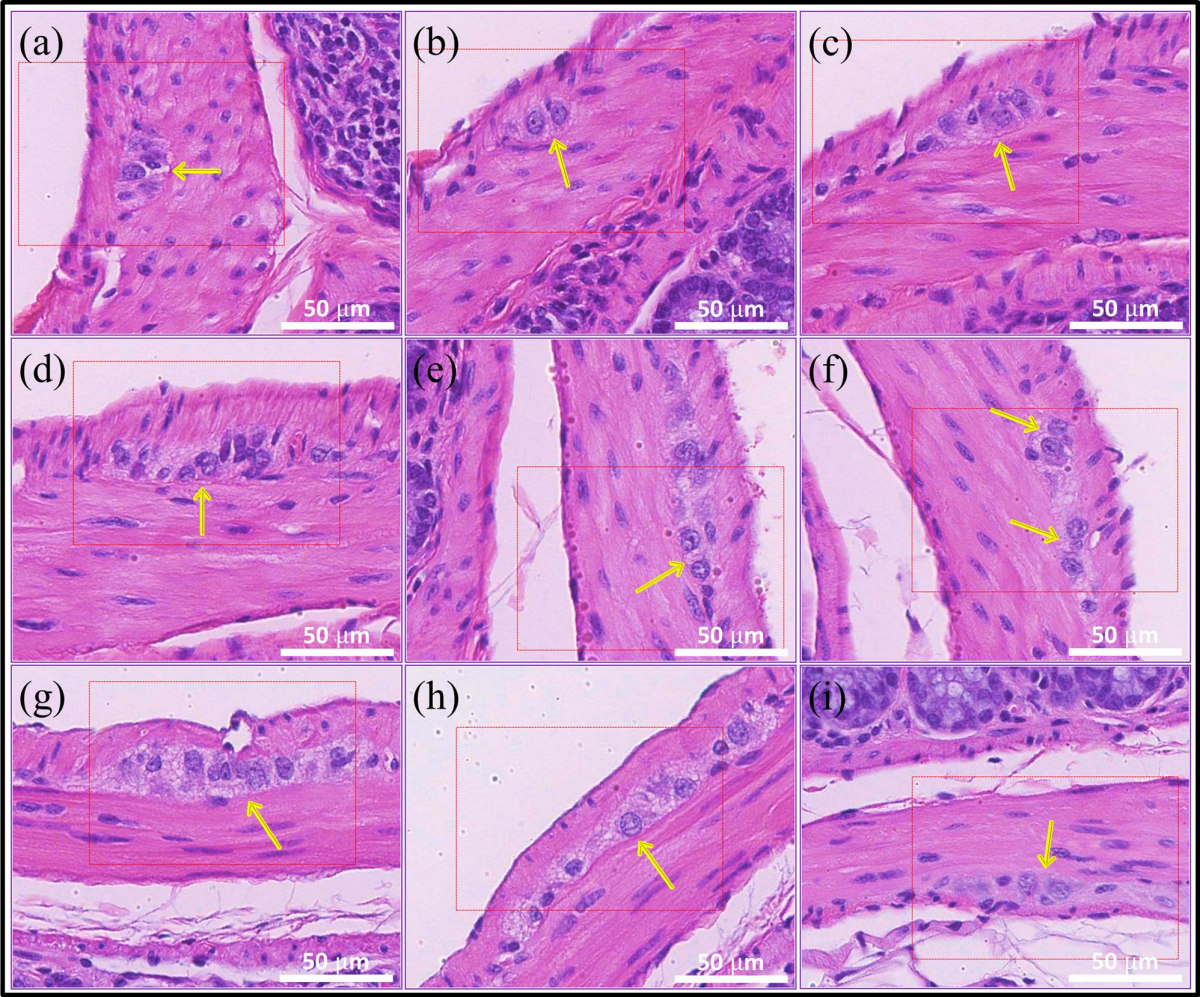
Brightfield images of H&E stained colon sections containing ganglion cells. Yellow arrows highlight some of the ganglion cells present.

Figure 3 shows images from H&E stained slides, where ganglion cells, located within the muscularis propria of the colon, were identified as those with large, round well-defined nuclei and abundant cytoplasm as per standard H&E histology (indicated by yellow arrows).

**Figure 4:**
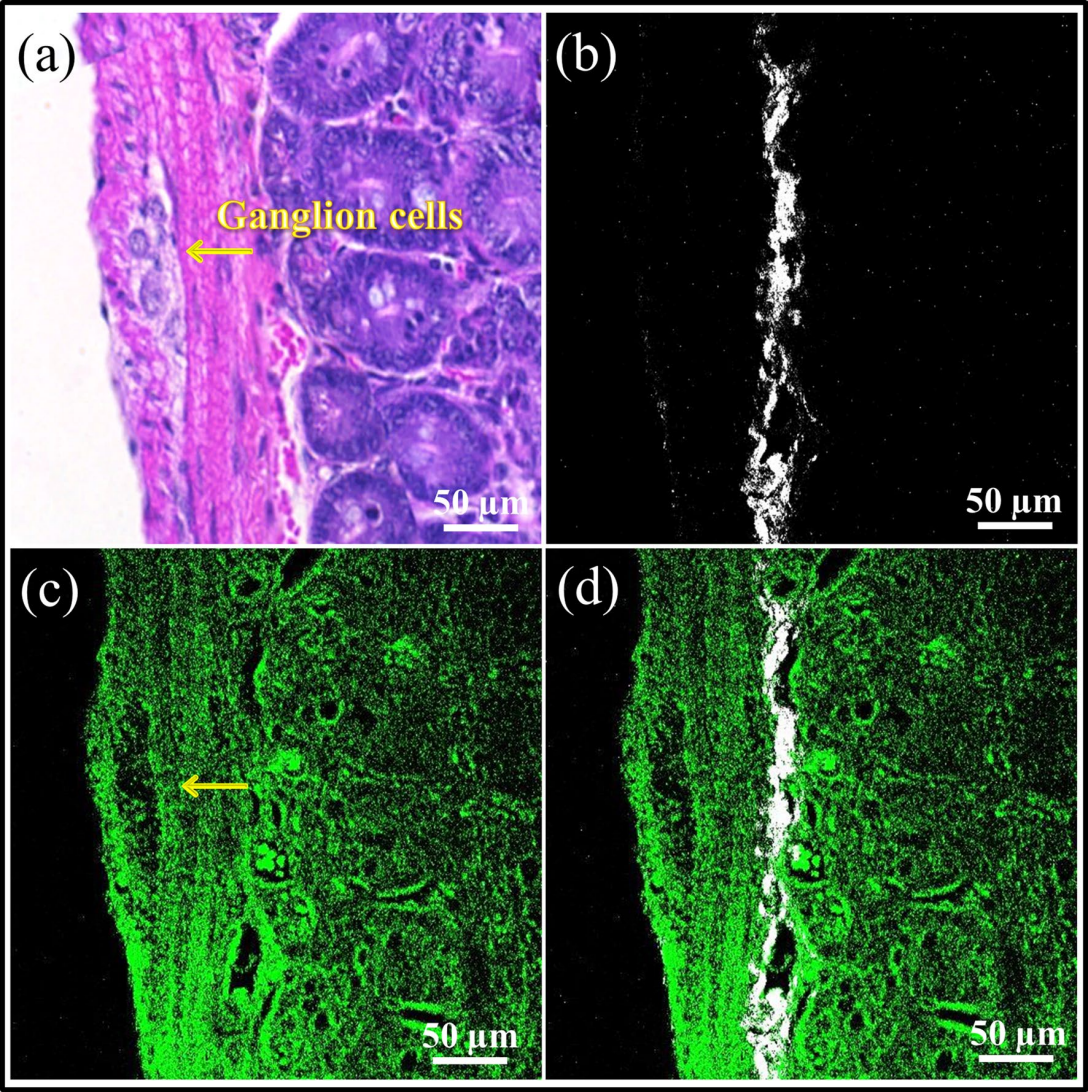
(**a**) Brightfield image from an H&E stained tissue section. Label-free (**b**) SHG, (**c**) 2PAF, and (**d**) overlaid SHG (white) and 2PAF (green) images from an adjacent near-identical section.

Figure 4 shows an H&E stained tissue section and its corresponding label-free SHG, 2PAF, and overlaid SHG/2PAF images from an adjacent unstained tissue section. The SHG image shows frequency doubled signals within the submucosa region, from a thin layer of connective tissue rich in collagen^22^, which is known to generate strong SHG signals^15^. This SHG signal, therefore, is a useful optical marker for initially determining the orientation of an unstained tissue section. The submucosa layer can be located, as well as the relative locations of the muscularis propria (containing ganglion cells) and the mucosa on either side. The 2PAF image provides additional morphological and structural information of the surrounding tissue. In general, regions containing ganglion cells showed lower autofluorescence signals compared to the adjacent tissue, resulting in these regions having a negative contrast in the 2PAF images. These results indicate that SHG/2PAF imaging is useful for rapidly surveying the tissue and locating regions of interest (ROIs) potentially containing ganglion cells. However, because other non-ganglion cell regions in the 2PAF image in Fig. 4c also exhibit a negative contrast, autofluorescence alone is insufficient to accurately identify ganglion cells due to its low specificity.

**Figure 5:**
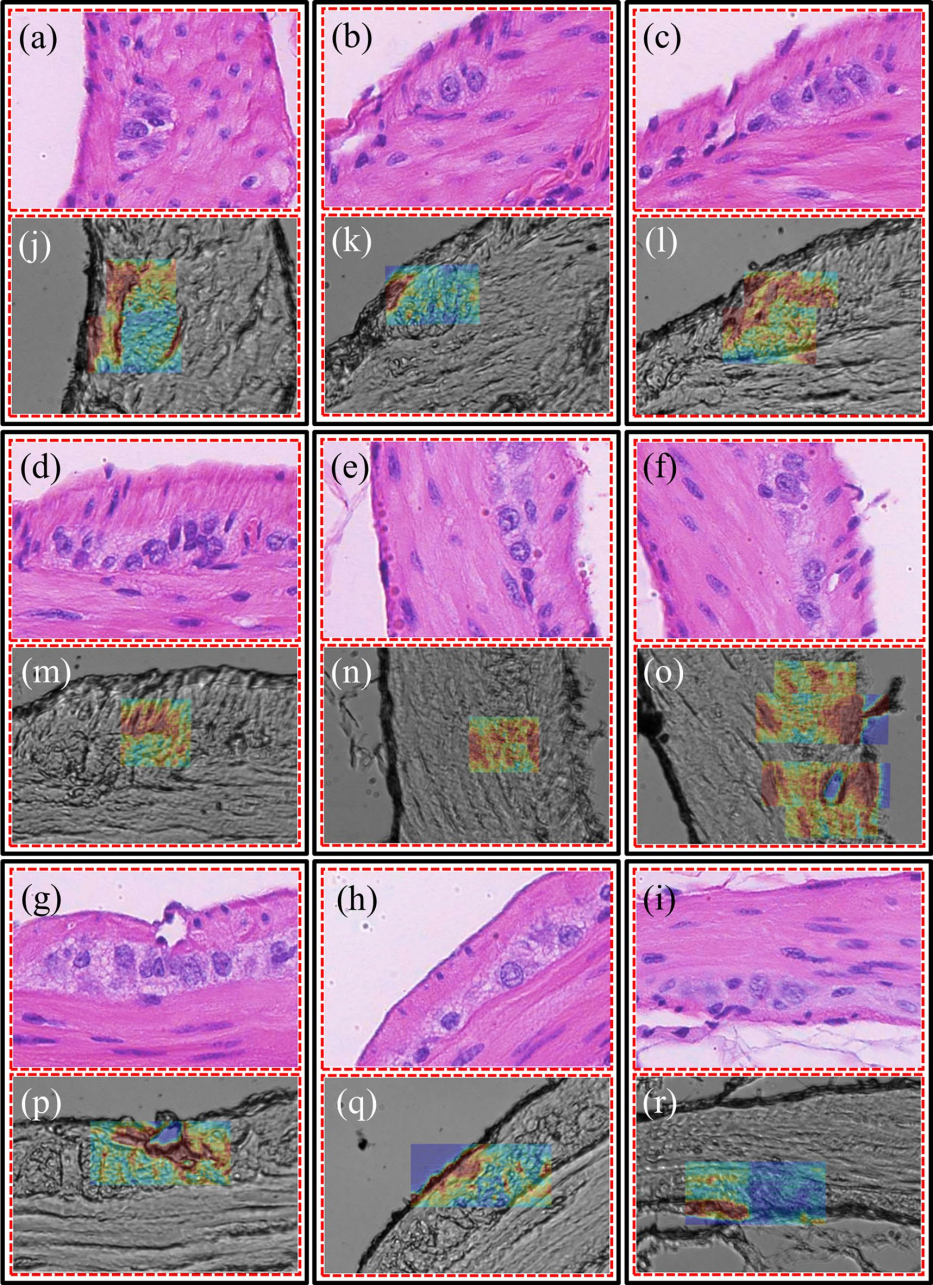
Brightfield images (**a**–**i**) from H&E stained areas, indicated by dashed red rectangles in Fig. 3, and (**j**–**r**) from the near-identical unstained areas with overlaid Raman imaging (by using the 1450 cm^−1^ peak) in areas containing ganglion cells.

Hyperspectral Raman images were acquired from the ganglion cells and surrounding tissue, as shown in Fig. 5j–r, and correlated to adjacent H&E sections (Fig. 5a–i) to determine if the multiplexed Raman spectral information has higher specificity for identifying ganglion cells.

**Figure 6:**
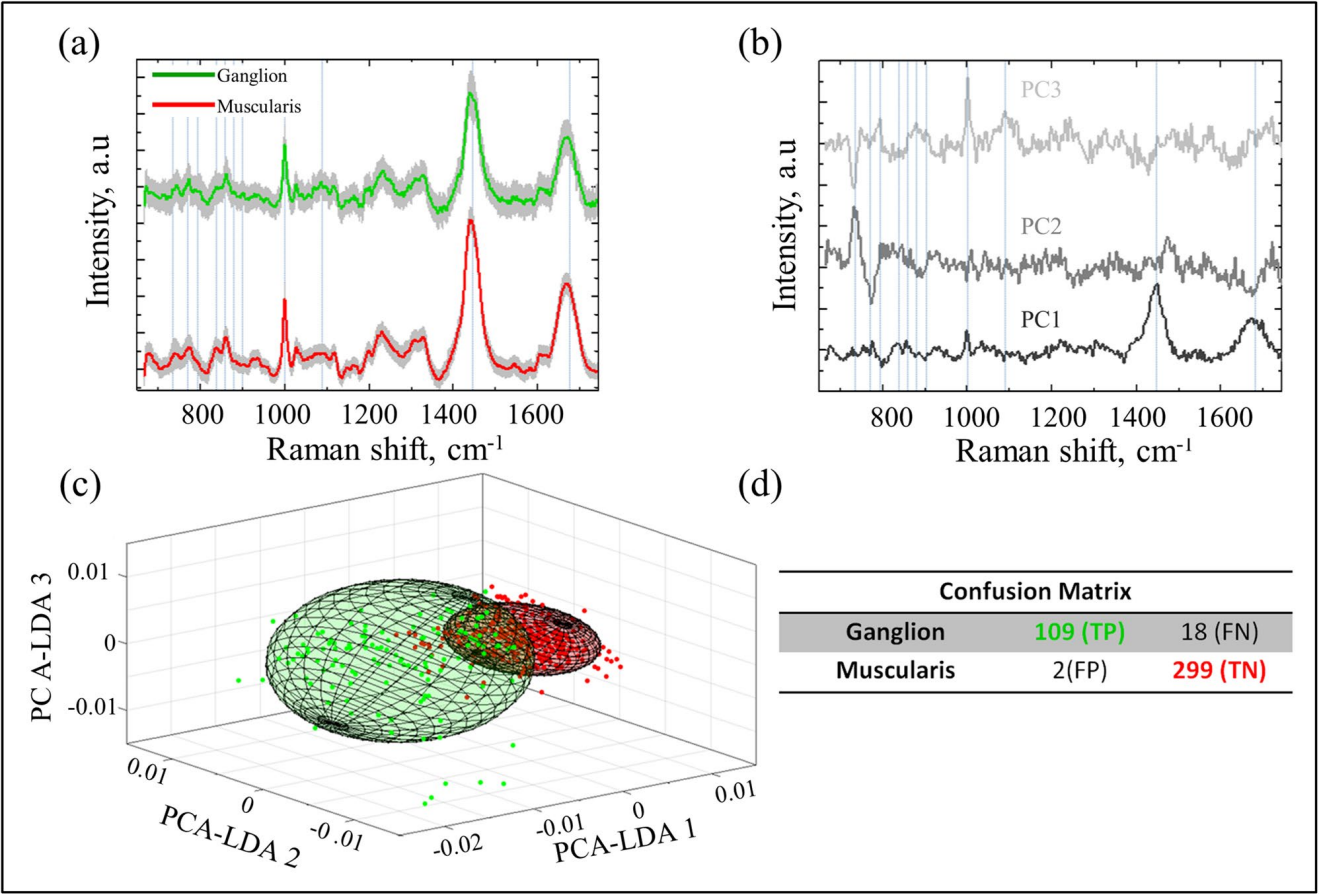
(**a**) Average Raman spectra of ganglion cells (green) and muscularis propria tissue (red), with the gray shadows representing ± 1 SD over the average values. (**b**) Plot of the first three PC coefficients, (**c**) 3D PCA-LDA plot with 90% interval confidence covariance error ellipsoids, and (**d**) confusion matrix generated by the classification model.

Raman fingerprints were extracted from a total of 428 different locations within ganglion cells (n = 127) and muscle tissue (n = 301) regions. Figure 6a shows the averaged Raman spectra for all 127 ganglion (green line) and 301 muscle tissue (red line) locations. The gray shadows represent ± 1 standard deviations (SD) over the average values. Figure 6b shows the coefficient plot of the first three PCs and highlights some Raman peaks that contribute most in discriminating the groups: 741, 770, 794, 837, 877, 901, 859, 1001, 1087, 1447, 1676 cm^−1^. We noticed that the ratio intensity between the 1447 and 1676 cm^−1^ peaks, assigned to lipids and proteins are very relevant in the discrimination. Also, compared to muscle layer, spectra from the ganglion cells have stronger peaks associated with nucleic acids (741 and 794 and 1087 cm^−1^) and lipids (770, 837 and 877 cm^−1^), which is in agreement with previous study^23^. A 3-D plot of the covariance error ellipsoids with 90% interval confidence, which determines the boundaries of the PCA-LDA prediction model, is shown in Fig. 6c. The accuracy of the prediction model, 95%, was quantified in the confusion matrix (Fig. 6d) with 86% sensitivity and 99% specificity.

**Figure 7:**
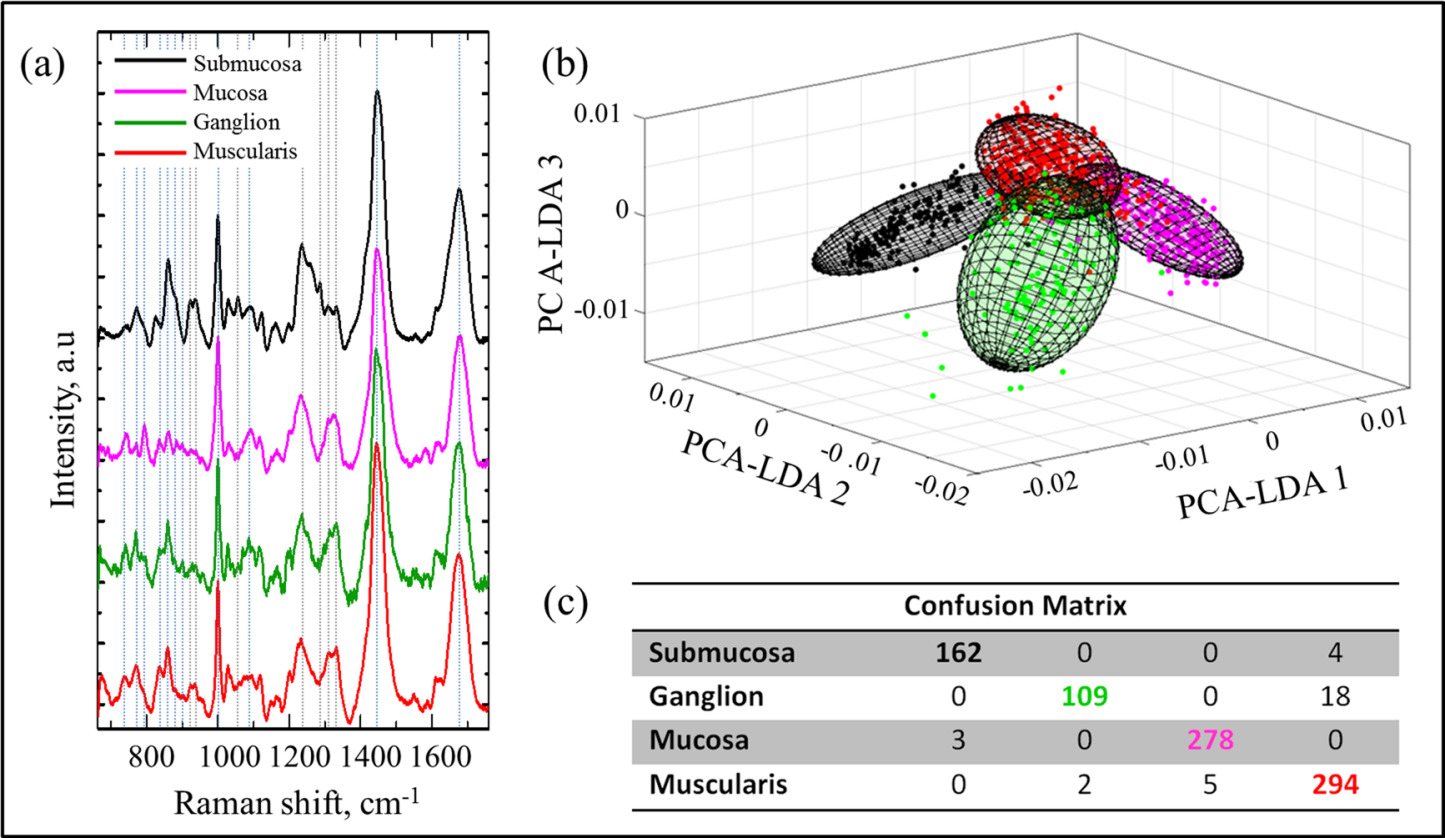
(**a**) Offset Raman average spectra from the 4 different groups. (**b**) 3D PCA-LDA plot with 90% interval confidence covariance error ellipsoids and (**c**) confusion matrix by using the classification model.

Raman fingerprints of the connective submucosa (n = 166) and the mucosa layers (n = 281) were also acquired. Figure 7a shows the average Raman spectra of each group. As expected, the submucosa region, which emit strong SHG signals due to the presence of collagen, exhibit strong Raman signals at 923, 938, 1056, 1235, 1286, 1309 and 1332 cm^−1^, all known Raman peaks associated with collagen^24^. Figure 7b,c shows the discrimination of all 4 groups together, with an overall accuracy of 96%. All of the misclassified ganglion cell spectra were assigned to the muscularis group, which is not surprising considering the close proximity of these two regions to each other.

**Figure 8:**
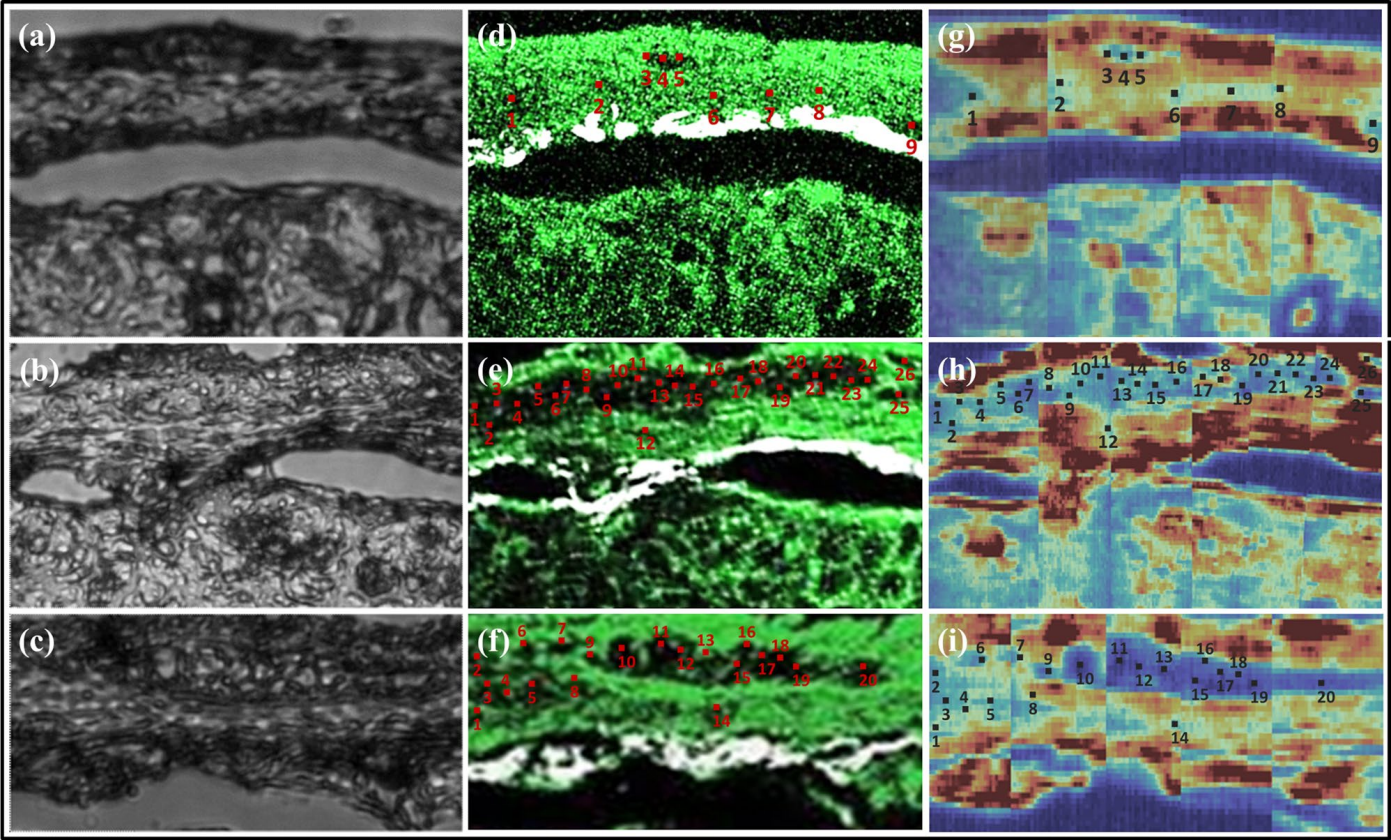
(**a**–**c**) Brightfield images from unstained section and their correspondent label-free images: (**d**–**f**) SHG and 2PAF overlaid signals; (**g**–**i**) Raman map by using the 1450 cm^−1^ peak.

**Figure 9:**
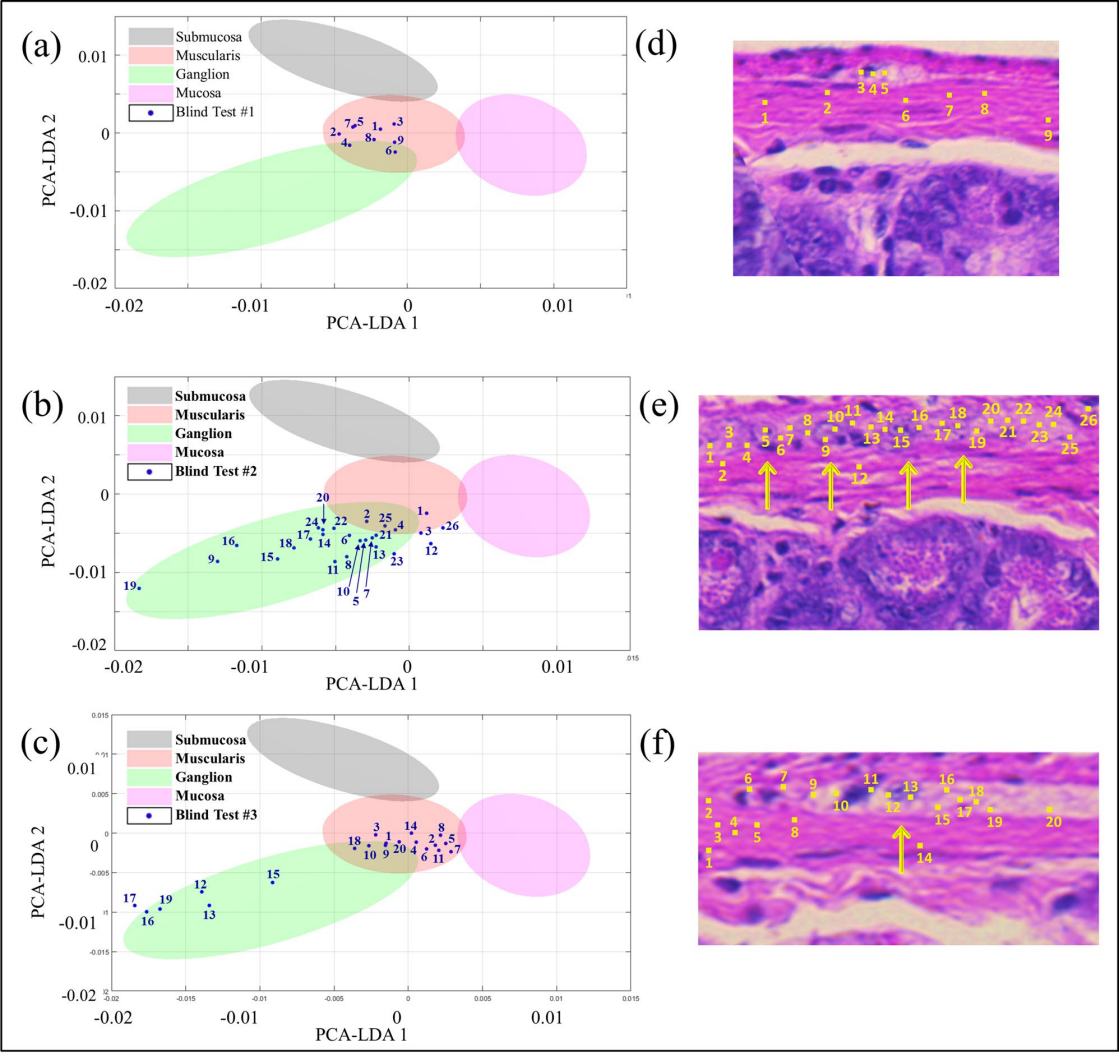
(**a**–**c**) 2-D PCA plot showing the classification of the Raman spectra acquired from different locations in the tissue specimens (blue dots) based on the PCA-LDA model (shadowed ellipses) defined by the previous Raman spectral data from Fig. 7. (**d**–**f**) H&E images showing the location of ganglion cells (yellow arrows) and the numbered locations where Raman spectra were acquired.

To mimic a more realistic diagnostic scenario, we performed blinded experiments on unstained tissue sections without prior knowledge of whether they contained ganglion cells. That determination was based solely on the label-free imaging results, which were then compared to H&E stained adjacent tissue sections at the end of the experiment. Figure 8 shows brightfield images of three unstained colon sections and their corresponding SHG/2PAF and hyperspectral Raman images. Using the SHG signal from the connective submucosa layer as an initial guide, the muscularis layer (above the SHG line) and the mucosa (below the SHG line) are identified. Within the muscularis layer, regions of negative 2PAF contrast that are potentially ganglion cells are located. Raman fingerprints were collected from these regions and adjacent regions above the connective submucosa layer, as depicted by the numbered locations in Fig. 8. PCA was then performed on this data set and the results were plotted on the PCA-LDA plot and classification model defined by the previous reference dataset (from Fig. 7). Figure 9 shows the PCA-LDA plot and the corresponding H&E images with yellow arrows highlighting the ganglion cells, as determined by a pathologist, and numbered locations corresponding to the positions where Raman spectra were acquired. Although the first specimen exhibited a negative 2PAF contrast (Fig. 8d) suggesting the presence of ganglion cells in that area, the Raman spectral data shows no ganglion cells present (Fig. 9a), which is confirmed by the pathologist through the H&E image (Fig. 9d). For the two other specimens, the Raman spectra indicate the presence of ganglion cells (Fig. 9b,c), which is confirmed by the H&E images (Fig. 9e,f).

As an alternative to the PCA-LDA classification, SVM was also performed on the data from the blinded experiments. Every Raman spectrum was a *1340*-dimensional vector that was classified and assigned to a group by using the previous Raman data as the training set. Table 1 shows that the results from the SVM are in good agreement with the PCA-LDA classification model and are consistent with the identification of ganglion cells from the H&E image analysis.

**Table 1:** SVM classification for the blind tests.

Observation	Blind test #1	Blind test #2	Blind test #3
1	‘Muscularis propria’	‘Muscularis propria’	‘Muscularis propria’
2	‘Muscularis propria’	‘Ganglion’	‘Muscularis propria’
3	‘Muscularis propria’	‘Muscularis propria’	‘Muscularis propria’
4	‘Muscularis propria’	‘Muscularis propria’	‘Muscularis propria’
5	‘Muscularis propria’	‘Ganglion’	‘Muscularis propria’
6	‘Muscularis propria’	‘Ganglion’	‘Muscularis propria’
7	‘Muscularis propria’	‘Ganglion’	‘Muscularis propria’
8	‘Muscularis propria’	‘Ganglion’	‘Muscularis propria’
9	‘Muscularis propria’	‘Ganglion’	‘Muscularis propria’
10		‘Ganglion’	‘Muscularis propria’
11		‘Ganglion’	‘Muscularis propria’
12		‘Muscularis propria’	‘Ganglion’
13		‘Ganglion’	‘Ganglion’
14		‘Ganglion’	‘Muscularis propria’
15		‘Ganglion’	‘Ganglion’
16		‘Ganglion’	‘Ganglion’
17		‘Ganglion’	‘Ganglion’
18		‘Ganglion’	‘Muscularis propria’
19		‘Ganglion’	‘Ganglion’
20		‘Ganglion’	‘Muscularis propria’
21		‘Muscularis propria’	
22		‘Ganglion’	
23		‘Muscularis propria’	
24		‘Ganglion’	
25		‘Muscularis propria’	
26		‘Muscularis propria’	

## Discussion

In this study, detailed experiments were performed to correlate label-free and H&E images of adjacent intestinal tissue sections to discover label-free SHG, 2PAF, and Raman optical signals that can be used to accurately locate and identify ganglion cells in unstained specimens. We found that SHG and 2PAF signals enabled the overall tissue morphology and structure to be visualized, and specifically allowed us to locate the submucosa layer and neighboring tissue region (muscularis) potentially containing ganglion cells. However, because the negative 2PAF contrast, alone, was insufficient for reliably identifying ganglion cell areas, we used hyperspectral Raman microscopy to acquire more spectral and chemical information. The results of two classification approaches, PCA-LDA and SVM, showed that Raman spectral markers exist for accurately identifying ganglion cells, when compared to conventional H&E histology.

Although specimens from wild type mice were used in this study, our results lay the foundation for establishing the optical signatures of ganglion cells and intestinal tissue that will be of paramount importance for future planned studies using tissue specimens from a knockout mouse model of Hirschsprung disease. Furthermore, animal models have been widely used and are useful resources for research in disease development, diagnostics and therapeutics study of human diseases^25^. In particular, several previous studies using mice models have contributed to improve the understanding of HD^26,27^. Future research will involve human patient specimens as we advance the technology towards clinical implantation.

We envision a near real-time intraoperative histology approach could be developed based on these three optical modalities integrated into a single system for diagnosing intestinal tissue biopsies in a clinical setting in which an unstained, unsectioned tissue biopsy obtained during surgery can be subjected to multimodal imaging and spectroscopy. First, SHG and 2PAF multiphoton imaging, with its high resolution optical sectioning capability, would be used to image the overall tissue architecture, to identify the tissue layers of interest and to pinpoint regions potentially containing ganglion cells. Then, the instrument would be switched to Raman spectroscopy to diagnose those regions as ganglion or non-ganglion. Given that hyperspectral Raman microscopy is a slow imaging method, it could be used to only interrogate those specific regions identified by SHG/2PAF, instead of using Raman to image larger tissue areas, which would be impractically slow. Alternatively, faster Raman methods such as CARS and SRS could potentially be implemented, provided that enough spectral markers can be acquired for accurate identification.
